# Bacteriocin-Producing Enterococci Modulate Cheese Microbial Diversity

**DOI:** 10.1007/s00248-025-02491-7

**Published:** 2025-01-22

**Authors:** Claudia Teso-Pérez, Areli López-Gazcón, Juan Manuel Peralta-Sánchez, Manuel Martínez-Bueno, Eva Valdivia, María Esther Fárez-Vidal, Antonio M. Martín-Platero

**Affiliations:** 1 Departamento de Microbiología, Universidad de Granada, Avda. Fuentenueva, S/N, 18071 Granada, Spain; 2 Departamento de Zoología, Facultad de Biología, Universidad de Sevilla, 41012 Seville, Spain; 3 Instituto de Biotecnología, Universidad de Granada, C/ Ramón y Cajal, 4, 18071 Granada, Spain; 4 Departamento de Bioquímica y Biología Molecular III e Inmunología, Facultad de Medicina, Universidad de Granada, 18016 Granada, Spain; 5 Instituto de Investigación Biomédica IBS. Granada. Complejo Hospitalario Universitario de Granada, Universidad de Granada, 18071 Granada, Spain

**Keywords:** *Enterococcus*, Cheese microbiota, Microbial interactions, Microbial diversity, Bacteriocins, Enterocins

## Abstract

Cheese production involves various lactic acid bacteria (LAB) that break down lactose, milk proteins, and fats, producing key nutrients and influencing the cheese’s flavor. They form communities that play a crucial role in determining the cheese’s organoleptic properties. The composition of cheeses’ microbial communities is shaped by physicochemical factors (e.g., temperature, pH, and salinity) and biological factors (i.e. microbial interactions). While starter cultures are introduced to control these communities, non-starter LAB represent a significant portion of the final microbial assemblage, but their interactions remain unclear. LAB often produce bacteriocins, antimicrobial peptides that antagonize other bacteria, but their role within LAB communities is not fully understood. This study aimed to assess the impact of bacteriocin production on LAB diversity in cheese, using *Enterococcus* as a model organism, a common bacteriocin producer. We analyzed enterocin production of enterococcal isolates by antimicrobial assays and microbial diversity differences in raw milk cheeses by two approaches: 16S RNA gene amplicon metagenomic sequencing for the whole microbial community and multi-locus sequence analysis (MLSA) for the enterococcal diversity. Our results revealed that LAB communities were dominated by lactococci, lactobacilli, and streptococci, with enterococci present in lower numbers. However, cheeses containing bacteriocin-producing enterococci exhibited higher microbial diversity. Interestingly, the highest diversity occurred at low levels of bacteriocin producers, but this effect was not observed within enterococcal populations. These findings suggest that bacteriocin production plays a key role in shaping LAB communities during cheese ripening, although further research is needed to understand its broader implications in other microbial ecosystems.

**Supplementary Information:**

The online version contains supplementary material available at 10.1007/s00248-025-02491-7.

## Introduction

Cheese is a dynamic ecosystem that is constantly influenced by external and internal factors, including milk composition, cheese-making methods, ripening conditions, and interactions among microbial communities. Multiple biochemical reactions and microbial interactions during the ripening process are involved in the development of desirable product sensory and organoleptic characteristics, including a pleasing taste and aroma and protection against spoilage, foodborne pathogens, and negative health effects [1, 2].

The microbial communities in cheese are highly complex, with lactic acid bacteria (LAB) being the most abundant microorganisms. LAB are a heterogeneous group of bacteria with the ability to ferment carbohydrates into lactic acid via homo fermentation, or into different end products (CO_2_, lactate and acetate, or ethanol) via heterofermentative metabolism [3, 4]. This rapid acidification of fermented milk inhibits the growth of the majority of undesirable microorganisms [5], thereby extending the shelf life of the fermented food. LAB also contribute to the flavor, texture, and nutritional value of the cheese by producing diacetyl, acetoin, acetaldehyde, acetic acid, and other organic acids, and a wide range of volatile compounds [6, 7]. Although the LAB naturally present in milk have traditionally been used in cheese-making, it is now more common to select fully characterized LAB strains as starter cultures to improve the consistency of fermentation [8]. Growth of the cheese microbiota is also controlled by regulating temperature, salt, and humidity levels [9]. Nevertheless, a secondary or adventitious LAB microbiota, known as non-starter lactic acid bacteria (NSLAB), spontaneously develops in cheese made with either pasteurized or raw milk, especially during ripening [4]. This microbiota does not usually contribute to acid production [10] but plays a major role in cheese ripening, influencing the final flavor and texture [11]. In particular, cheese made with raw milk usually has a complex microbiota characterized by the succession of different microorganisms throughout the cheesemaking process, from starter LAB to NSLAB [12]. This succession of communities may be influenced by the ripening conditions and by dynamic interactions among the microorganisms [1], which can be beneficial, neutral, or harmful, and affect the outcome of cheese ripening.

Antagonism is a frequent type of interaction in dairy fermentation, either through the production of acids via metabolism of carbohydrates, the presence of bacteriophages, or the production of antimicrobial peptides such as bacteriocins [1]. Bacteriocins are ribosomal synthesized antimicrobial peptides that kill or inhibit the growth of closely related bacteria [13, 14]. They are considered to be safe and natural preservatives with immense potential for utilization alone or alongside other techniques in food preservation [15]. Bacteriocin-producing strains commonly have self-protection mechanisms against their own bacteriocins, allowing their possible utilization as “protective cultures” in cheese to inhibit the growth of pathogens and spoilage microorganisms [16]. The fact that a large majority of LAB produce bacteriocins [15, 17] contrasts with the high diversity of the microbial communities, raising questions about their ecological function.

In studies on intertidal marine predators, Paine [18] was the first to propose that negative interactions favor greater biodiversity by preventing one species from outcompeting others. A similar effect has been proposed for antagonistic interactions mediated by bacteriocins in bacterial communities, where bacteriocins may also play an important role in promoting biodiversity by producing ecological units of producers and resistant strains [19]. These ecological units could arise from the dynamics between producing, resistant, and sensitive strains in a similar manner to the game of “rock-paper-scissors.” In this way, the toxin producer can kill the toxin-sensitive strain, the toxin-sensitive strain can outgrow the toxin-resistant strain, and the toxin-resistant strain can outgrow the toxin-producing strain [20]. This model can be extended to multiple populations if they form units of mutually immune strains that act as a single entity in this rock-paper-scissors game against each other [21].

Given this background, the objective of this study was to examine the effect of bacteriocin producer populations on community diversity. To this end, we used *Enterococcus* as a model organism because there are frequent bacteriocin producers in this genus, and its population levels in cheese are variable, ranging from 10^3^ to 10^8^ CFU/g in ripened cheese [22, 23]. This allows to study the effect of enterocin producers under different diversity scenarios on cheese, which is an excellent model system for studying the mechanistic principles that drive microbial diversity [24, 25]. Results obtained indicate a positive effect of enterocin production by enterococcal populations on the overall LAB community biodiversity when bacteriocin-producer populations are small. These findings shed light on the impact of antimicrobial peptide production as a driver of LAB biodiversity.

## Material and Methods

### Sampling and Microbial Enumeration

Given the higher diversity of raw milk cheeses [2], the study included 15 different commercial ripened raw milk cheeses (Supplementary Table S1): five from cow’s milk, five from sheep’s milk, and five from goat’s milk. Cheese samples (5 g) were homogenized for 2 min in 45 mL of a prewarmed (37 °C), sterile, 2% sodium-citrate solution in sterile plastic bags with lateral filters using a masticator lab blender (IUL Instruments, Barcelona, Spain). Tenfold serial dilutions of this homogenate were then prepared in sterile 2% sodium-citrate solution up to a dilution of 10^–6^. A 100 μL aliquot was spread in triplicate on agar plates for bacterial enumeration, using Brain Heart Infusion agar (BHI, VWR Chemicals) for total LAB, and Kenner Fecal agar (KF, VWR Chemicals) for enterococci. Viable counts were obtained after incubation for 3 days at 28 °C for total LAB enumeration and 37 °C for enterococci enumeration. Bacterial counts were calculated as the mean values of three determinations after log transformation.

### Antimicrobial Assays and Microbial Isolation

The abundance of enterocin producers was estimated by testing randomly 45–52 enterococcal colonies per sample (KF medium) for their antimicrobial capacities, employing the double-layer plate method of Gratia and Fredericq [26]. Enterococcal colonies were transferred by toothpick onto BHI agar buffered (BHA-B) in 0.1 M sodium phosphate buffer at pH7 (in triplicates) and incubated at 37 °C overnight. Next day, plates were overlaid with 5 mL of BHA-B soft agar (1.8% BHI, 0.8% agar) inoculated at 2% with an overnight culture of the indicator strains *Listeria innocua* (CECT4030) or *Enterococcus faecalis* S-47 (lab collection) and left to solidify at room temperature. Plates were incubated again overnight at 37 °C for the growth of the indicator strain. Colonies with an evident inhibition zone around the strain spot were considered to be enterocin producers. Subsequently, a representative set of 67 isolates were collected (5–7 from each sample) for further analysis, selected according to their zone of antibacterial activity. Pure cultures were stored in BHI medium containing 20% glycerol at − 80 °C.

To evaluate cross-immunity between selected isolates, all strains were tested against each other using the aforementioned double layer plate technique. Plates were incubated again overnight at 37 °C for growth of the indicator strain, and colonies with an evident inhibition zone around the strain spot were considered to be enterocin producers.

### DNA Extraction

Enterococcal populations and microbial communities were characterized by extracting total DNA from each strain and each cheese sample, following the modification of the salting-out procedure (MSOP) of Martin-Platero et al. [27]. Briefly, cells from 1 mL of an overnight culture of each isolate or cells from 2.5 g of cheese were resuspended in 100 µL of lysozyme buffer and incubated for 30 min at 37 °C to hydrolyze the cell wall, followed by the addition of 600 µL of lysis buffer and a further incubation step at 80 °C to facilitate cell lysis. Cell debris and proteins were then removed by adding 200 µL of protein precipitation solution, mixing, chilling on ice for 10 min, and centrifuging for 10 min at 20,000 × *g*. DNA was then precipitated with an equal volume of isopropanol and washed with 70% ethanol. The DNA was finally dried to remove any ethanol trace and dissolved in 200 µL of 0.5 × Tris–EDTA buffer (Tris-ClH 10 mM pH 8, EDTA 1 mM). DNA concentrations were measured using a NanoDrop 2000 spectrophotometer (Thermo Fisher Scientific).

### Multi-Locus Sequence Analysis (MLSA)

Representative bacterial isolates (67 strains; 5–7 from each sample) were characterized by MLSA to estimate the population variability of the genus *Enterococcus* in each cheese sample. To this end, we constructed a phylogenetic tree based on the concatenated sequences of five housekeeping genes (Table 1): *adk* (adenylate kinase), *atpA* (ATP synthase, alpha subunit), *gyd* (glyceraldehyde-3-phosphate dehydrogenase), *gdh* (glucose-6-phosphate dehydrogenase) [28], and *groEL* (chaperone GroEL). Primers for *groEL* gene were designed using Hyden software [29], allowing for two mismatches and two degenerate positions and targeting an amplicon size between 400 and 600 bp.

**Table 1:** Primers used for MLSA analysis

Primer pair	Gene	Primer sequence (5′- > 3′)	Amplicon size (bp)	Reference
adk adk2	Adenylate kinase	TATGAACCTCATTTTAATGGG GTTGACTGCCAAACGATTTT´	437	[28]
atpA1 atpA2	ATP synthase, alpha subunit	CGGTTCATACGGAATGGCACA AAGTTCACGATAAGCCACGG	556	
gyd1 gyd2	Glyceraldehyde-3-phosphate dehydrogenase	CAAACTGCTTAGCTCCAATGGC CATTTCGTTGTCATACCAAGC	395	
gdh1 gdh2	Glucose-6-phosphate dehydrogenase	GGCGCACTAAAAGATATGGT CCAAGATTGGGCAACTTCGTCCCA	530	
GroELF GroELR	Chaperone GroEL	GYGAAAAATTWCAAGAACG ACGACWGCTTCAGTYGTTAA	480	This study

Each gene for each bacterial isolate was PCR-amplified using the following conditions: an initial denaturing step of 94 °C for 3 min followed by an amplification step of 35 cycles of 30 s at 94 °C, 30 s at 50 °C, and 30 s at 72 °C, with a final extension of 5 min at 72 °C. Reactions were performed in a final volume of 50 µL with Taq polymerase (1x) and buffers from IBIAN Technologies. Primers were used at a concentration of 0.5 µM, and 100 ng of DNA was added. PCR products were purified with the NucleoFast 96 PCR clean-up kit (Macherey–Nagel) and sequenced with PCR forward primers by Sanger sequencing [30], using the Stab Vida (Universidad de Nova de Lisboa) sequencing service. Next, each sequence was taxonomically assigned according to the best blast hit on the NCBI blast tool (https://blast.ncbi.nlm.nih.gov/ Blast.cgi) [31].

Finally, a phylogenetic tree was constructed from the concatenated sequences. First, each gene was aligned and trimmed with MEGA-X software (version 10.1.8) [32] using the clustalW algorithm [33]. Then, the concatenation of the five genes was used to construct a maximum likelihood phylogenetic tree in MEGA-X with 1000 bootstrap replications following the Tamura-Nei evolution model (ML heuristic method: nearest-neighbor-interchange, number of threads: 3) [34].

### Data Analysis

#### 16S rRNA Metagenomic Sequencing

The microbial composition of cheese samples was determined by constructing and sequencing 16S rRNA metagenomic libraries for each sample. We constructed 16S rRNA libraries corresponding to the V4 variable region by a two-step amplification approach. The first primer pair (Mi_U515F, 5′- TCGTC GGCAG CGTCA GATGT GTATA AGAGA CAGGT GCCAG CMGCC GCGGT AA −3′ and Mi_E786R, 5′- GTCTC GTGGG CTCGG AGATG TGTAT AAGAG ACAGG GACTA CHVGG GTWTC TAAT −3′) contained the primer sequences of U515F and E786R targeting the V4 region of the 16S rRNA gene with partial overlap of Illumina primers, as previously described [35]. PCR was carried out in a final volume of 25 µL containing 12.5 µL of Phusion Flash High-Fidelity PCR Master Mix (Thermo Scientific™), 0.3 µM of each primer, and 5 µL of template DNA. The amplification program comprised an initial denaturing step of 98 °C for 10 s followed by an amplification step of 20 cycles of 1 s at 98 °C, 5 s at 52 °C, and 5 s at 72 °C, with a final extension of 1 min at 72 °C. PCR was purified using the MEGAquick-spin™ Plus Total Fragment DNA Purification Kit (iNtRON Biotechnology) and was re-amplified in a second PCR to introduce a unique combination of two barcodes for each sample. This PCR was performed in a final volume of 25 µL containing 12.5 µL of Phusion Flash High-Fidelity PCR Master Mix (Thermo Scientific™), 0.4 µM of each primer, and 5 µL of the purified PCR product from the first PCR. The amplification conditions were an initial denaturation step of 98 °C for 10 s followed by an amplification step of 9 cycles of 1 s at 98 °C, 5 s at 55 °C, and 5 s at 72 °C, with a final extension of 1 min at 72 °C. This second PCR was purified again using the aforementioned kit. Next, the DNA concentration was measured using a Qubit® 3.0 Fluorometer (Invitrogen, Carlsbad, CA, USA) and normalized to the same concentration. High-throughput sequencing was carried out on an Illumina MiSeq platform at the Scientific Instrumental Center of the University of Granada (CIC-UGR, Spain). Sequences were uploaded to the Sequence Read Archive (SRA) on the Genbank—NCBI webpage (https://www.ncbi.nlm.nih.gov/sra) under the PRJNA988471BioProject (BioSamplesSAMN36020620 to SAMN36020683).

#### Sequences and Data Analysis

QIIME2 2021.11 was used to process the 16S Illumina reads [36]. First, primers were trimmed with the cut adapt plugin [37], discarding untrimmed sequences. Then, amplicon sequence variants (ASVs) were inferred from paired reads by the dada2 plugin [38]. Given the expected insertion size of 253 bases for the construction, ASVs shorter than 245 bases were filtered out. Next, all ASVs were aligned by the *mafft* method [39] and used to construct a phylogeny. The representative sequences were taxonomically classified using the Silva-138 database clustered at 99% similarity [40] by the classify-sklearn method [41].

Differences in bacterial diversity were explored by estimating alpha diversity from the ASV richness, the Shannon diversity index [42], and Faith’s phylogenetic diversity (PD) [43] in QIIME2 using a rarefied table at a sequencing depth of 17,000 sequences per sample. We constructed general linear models (GLM) with the diversity indices as dependent variables and the presence of *Enterococcus*-bacteriocin producers as fixed factor. Parametric tests were applied, because all variables followed a Gaussian distribution (Shapiro–Wilk’s test of normality, *p* > 0.05).

In addition, the effect of antimicrobial strains on enterococci population diversity was determined by using Faith’s PD to estimate the alpha diversity [43], quantifying this index by using a phylogenetic tree inferred from MLSA results. We also calculated the population richness in each cheese sample by assigning each strain with a sequence type (ST), which was determined by clustering unique sequences of each gene using the USEARCH program (version 11.0.66; identity = 100%) [44]. PHYLOViZ software (version 2.0) was used to identify strains with the same allelic profile [45]. Strains grouped in the same cluster in four out of the five genes were considered the same population. The non-parametric Kruskal–Wallis test was applied to explore the relationship between the diversity indices and the presence of *Enterococcus*-bacteriocin producers, because none of the indices followed a Gaussian distribution (Shapiro–Wilk’s test of normality, *p* > 0.05).

Pearson’s correlation test was also applied to evaluate the relationship between the proportion of enterococci producers and the corresponding alpha diversity. Finally, the Student’s *t*-test for dependent variables was used to establish the significance of differences in sensitivity between strains from the same cheese and those from different cheeses. Statistica 10.0 [46] was used for all statistical analyses.

## Results

### Differential Diversity Among Samples

**Fig. 1:**
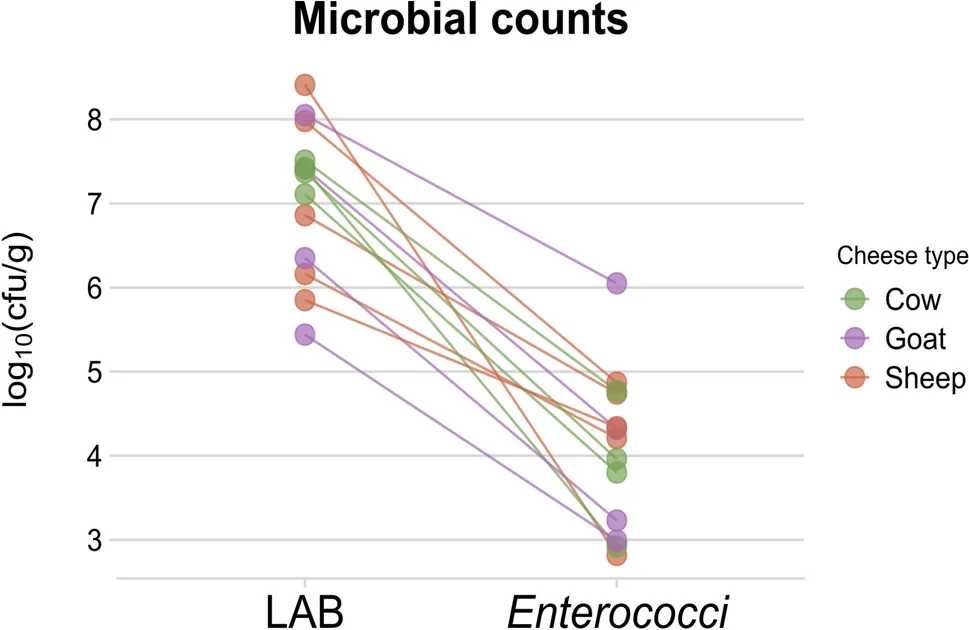
Cheese microbial counts. Total LAB counts and their corresponding *enterococci* counts are shown in logarithmic scale. Total LAB counts ranged from 5.4 to 8.4 log10 (cfu/g), while enterococci counts were on average threefold lower than total LAB counts; no growth of enterococci was observed in two samples. Colors indicate milk source

Microbial counts and compositions varied widely among the studied LAB communities (*n* = 15). The total LAB microbial count ranged from 2.8 × 10^5^ to 2.6 × 10^8^ CFU/g (Supplementary Table S1; Fig. 1), and the enterococcal count ranged from 6.6 × 10^2^ to 1.1 × 10^6^ CFU/g (Supplementary Table S1; Fig. 1) except in two samples that showed no growth (not depicted in Fig. 1). Hence, enterococcal populations were on average threefold lower than total LAB populations.

**Fig. 2:**
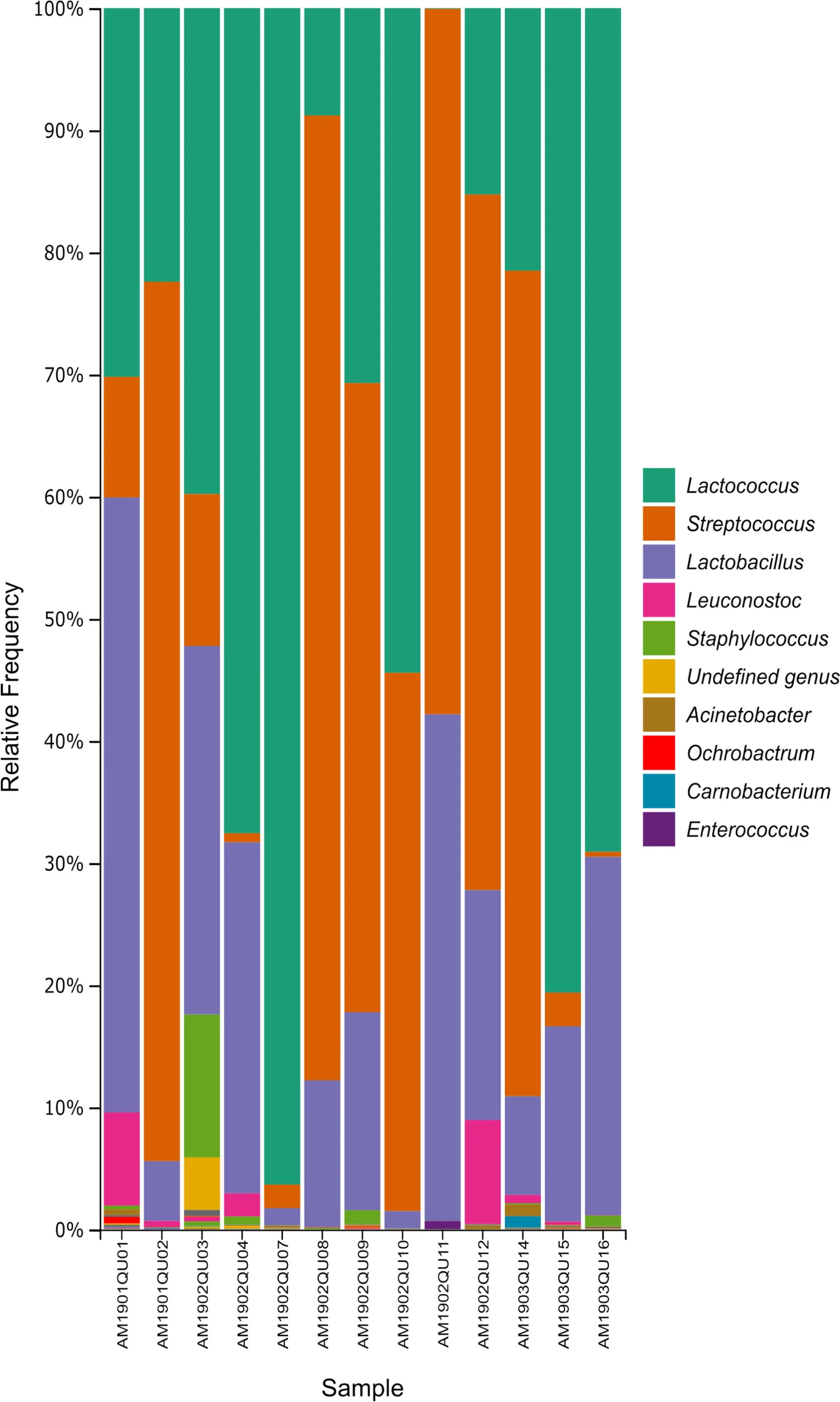
Bacterial genera present in raw milk cheeses. In all samples, the most abundant genera were *Lactococcus*, *Streptococcus*, *Lactobacillus*, and *Leuconostoc*. Color legend shows the ten most abundant genera overall ranked from most to least abundant

According to the corresponding 16S rRNA metagenomes, LAB communities were dominated by lactococci and streptococci*,* followed by lactobacilli, at proportions that varied among samples (Table 2 and Fig. 2). In all samples, populations of enterococci were four to five orders of magnitude lower than those of lactococci or streptococci, including samples where enterococci did not grow in their selective media (KF). The total number of ASVs ranged from 12.83 to 53.53, with the ranking for enterococci ranging from 8 to 40th position (average of 24th position) according to their abundance in the samples. Alpha diversity scores ranged from 0.1593 to 3.4817 for the Shannon index and from 1.190 to 3.360 for Faith’s PD.

**Table 2:** The ten most abundant bacterial genera per cheese according to 16S rRNA metagenomic analysis. All values are expressed as percentages

Cheese sample	Lactobacillus	Lactococcus	Streptococcus	Leuconostoc	Enterococcus	Acinetobacter	Staphylococcus	Ochrobactrum	Pseudomonas	Chryseobacterium
AM1901QU01	50.42	30.17	9.88	7.67	0.56	0.45	0.25	0.14	0.13	0.11
AM1901QU02	4.71	22.28	72.36	0.43	0.05	0.00	0.00	0.01	0.00	0.00
AM1902QU03	31.80	41.77	12.74	0.00	0.08	0.00	12.05	0.51	0.03	0.00
AM1902QU04	28.61	67.70	0.75	1.91	0.05	0.00	0.61	0.05	0.01	0.20
AM1902QU05	4.24	1.25	93.98	0.00	0.07	0.09	0.00	0.04	0.00	0.00
AM1902QU07	1.32	96.43	1.95	0.00	0.04	0.11	0.03	0.05	0.00	0.02
AM1902QU08	12.06	8.60	79.12	0.05	0.00	0.01	0.00	0.02	0.01	0.00
AM1902QU09	15.96	30.94	51.49	0.00	0.01	0.03	1.23	0.05	0.00	0.00
AM1902QU10	1.37	54.03	44.51	0.01	0.00	0.01	0.03	0.02	0.00	0.00
AM1902QU11	41.12	0.04	58.17	0.00	0.01	0.00	0.00	0.01	0.00	0.00
AM1902QU12	19.17	14.96	57.17	8.27	0.01	0.00	0.03	0.08	0.00	0.00
AM1903QU14	8.20	21.06	67.96	0.72	0.01	0.91	0.09	0.03	0.05	0.01
AM1903QU15	16.00	80.59	2.74	0.34	0.12	0.09	0.01	0.04	0.00	0.00
AM1903QU16	29.64	68.91	0.37	0.04	0.03	0.01	0.81	0.12	0.01	0.00
AM1903QU17	0.50	98.87	0.09	0.00	0.00	0.12	0.00	0.02	0.06	0.11

**Fig. 3:**
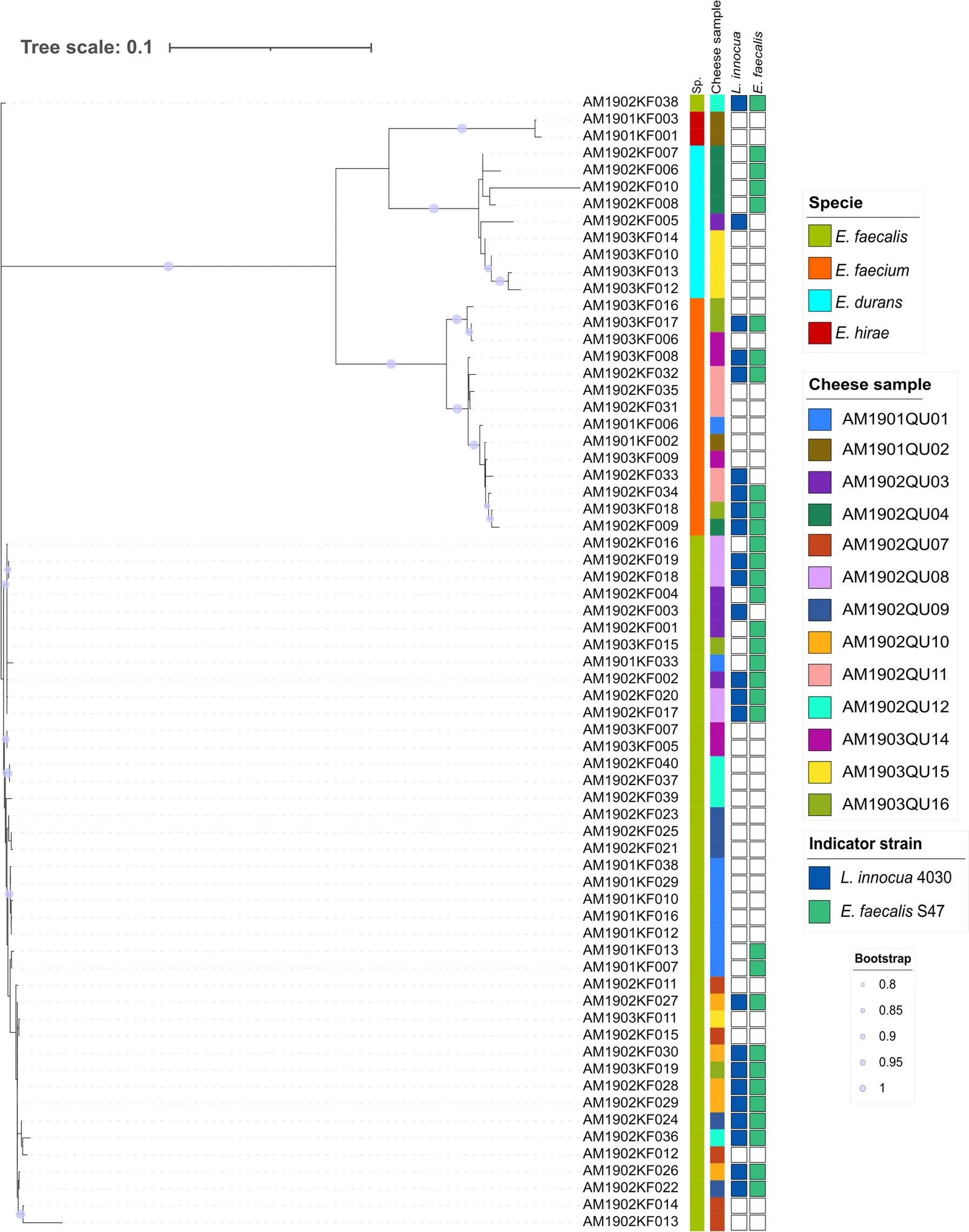
Phylogenetic tree of *Enterococcus* strains isolated from cheese made from raw milk. The phylogenetic tree is based on multi-locus sequence analyses (MLSA). From left to right, the first annotation bar represents the species: *E. faecalis* (green), *E. faecium* (orange), *E. durans* (blue), and *E. hirae* (red); the second bar represents the cheese sample and the third and fourth bars represent activity against *L. innocua* and *E. faecalis* S47, respectively

MLSA identified four species of enterococci present in our cheese samples: *E. faecalis*, *E. faecium*, *E. durans,* and *E. hirae* (Fig. 3). *E. faecalis* and *E. faecium* were the most frequently isolated species. The phylogeny inferred from MLSA results showed no homogenous clusters in relation to the sample or antimicrobial activity, indicating that the isolated enterococci were not part of a clonal population. In the case of *E. faecalis*, however, the genetic distance between strains was very short, with low bootstrap values.

Nevertheless, determination of enterococcal population richness by the assignation of type sequences yielded 57 different sequence types, observing 2–7 different type sequences in each cheese sample.

### Differential Antagonistic Producer Frequency Among Enterococci

**Fig. 4:**
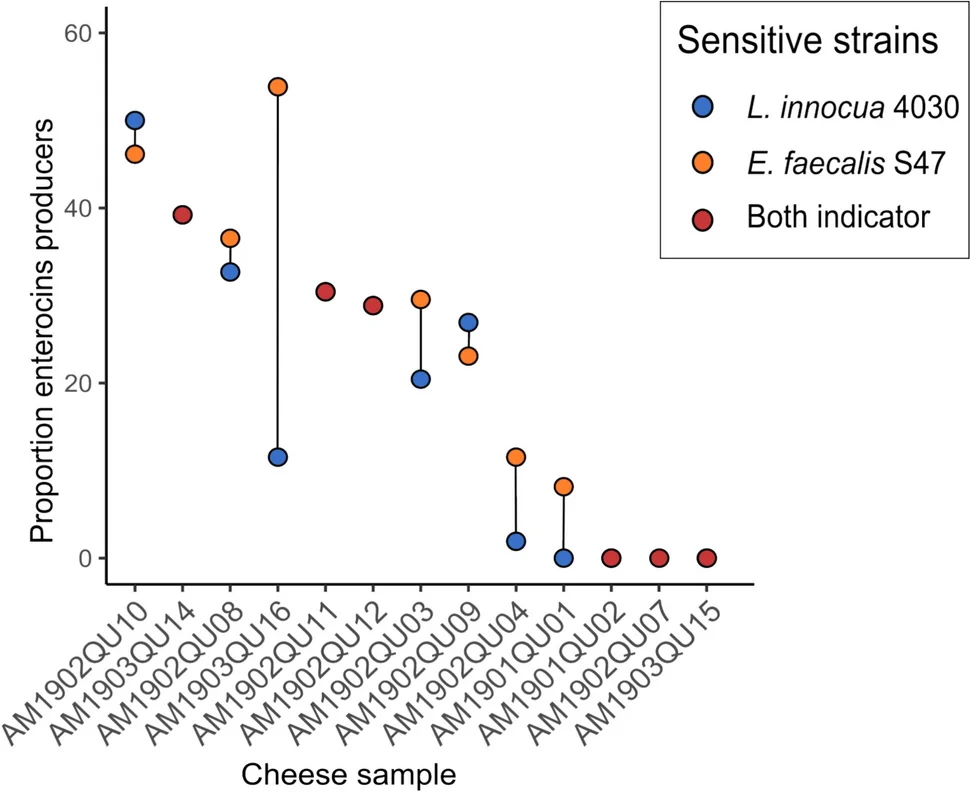
Enterocin producers. Proportion of enterocin producers with antimicrobial activity against *E. faecalis* S-47 (yellow circles), *L. innocua* CECT4032 (blue circles), and both indicator strains (red circles)

Between forty-four and fifty-two bacterial strains from each cheese were screened for antimicrobial activity, finding activity against *E. faecalis* S-47 in 23.64% of strains, activity against *L. innocua* in 18.5%, and activity against both in 21.25% (Fig. 4 and Table 3). Only three cheese samples had no strains with antimicrobial activity.

**Table 3:** Percentage of enterocin producers per sample

Cheese sample	% enterocin producers against *E. faecalis* S47	% enterocin producers against *L. innocua*
AM1901QU01	8.16	0
AM1901QU02	0	0
AM1902QU03	29.54	20.45
AM1902QU04	11.54	1.92
AM1902QU07	0	0
AM1902QU08	36.54	32.69
AM1902QU09	23.08	26.92
AM1902QU10	46.15	50
AM1902QU11	30.43	30.43
AM1902QU12	28.85	28.85
AM1903QU14	39.2	39.2
AM1903QU15	0	0
AM1903QU16	53.8	11.5

### Impact of Enterocin Producers on Population and Community Diversity

*Enterococcus* diversity was not significantly affected by the presence of enterocin producers in enterococcal populations. Given the lack of sharp clusters in the MLSA (Fig. 3), we estimated the population richness and used the phylogenetic tree inferred from MLSA results to quantify Faith’s PD [43] in each sample. Neither the population richness estimation nor Faith’s PD significantly differed between producers and non-producers (Table 4).

**Table 4:** Effect of enterocin-producing populations on enterococcal diversity. Kruskal–Wallis non-parametric test to evaluate effects of the presence/absence of *Enterococcus*-bacteriocin producers on alpha diversity indices of the enterococci populations

	*H*	*N*	*p*
Faith’s phylogenetic diversity index	1.02	13	0.31
Population richness	0.15	13	0.69

**Fig. 5:**
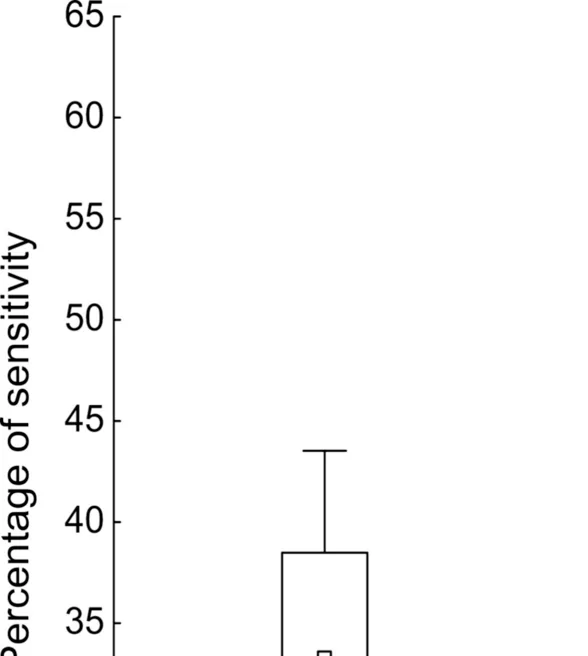
Boxplot showing the difference in sensitivity between enterococci in the same cheese sample and those in different cheese samples

Regarding the cross-immunity of each isolate (Supplementary Table S2), the frequency of inhibition was lower for enterococci that were present in the same cheese sample than for those that were not (Fig. 5). This difference was not statistically significant, although it was close-to-significant (*p* = 0.068) (Table 5).

**Table 5:** Student’s *t*-test of dependent variables to compare sensitivity between enterococci from the same cheese and those from different cheeses

	d.f	*N*	*p*
Same cheese * different cheeses	1.02	13	0.068

*d.f.* degree of freedom

**Fig. 6:**
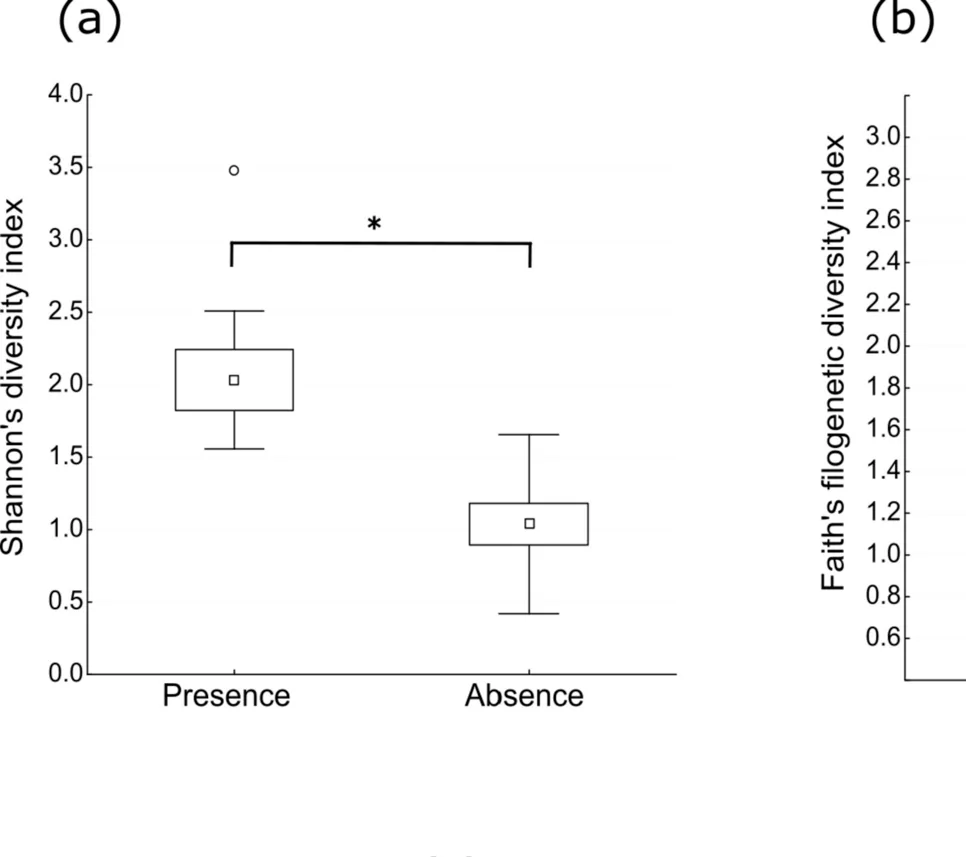
Boxplot showing differences in Shannon index (**a**), Faith’s phylogenetic diversity (**b**), and amplicon sequence variant richness (**c**) between raw milk cheeses with and without the presence of *Enterococcus*-bacteriocin producers. Dots represent the average, boxes the standard error of the mean, and whiskers the confidence interval. *A significant difference

**Fig. 7:**
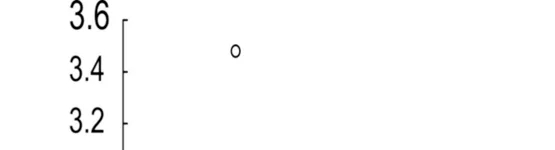
Correlations between proportion of *Enterococcus*-bacteriocin producers against *E. faecalis* S47 and the Shannon diversity index for the bacterial communities of cheeses

In contrast, the presence of enterocin producers increased the diversity of the whole community, being associated with a close-to-significant increase in ASV richness and Faith’s PD and with a statistically significant (*p* < 0.05) increase in Shannon index score (Table 6 and Fig. 6). This relationship between greater diversity and the presence of enterocin producers was explored by testing the correlation between these variables in the samples that contained enterocin producers. A negative relationship was observed between diversity and enterocin producers in these samples (Tables 7 and 8), but statistical significance was only reached for the Shannon index with *E. faecalis* as indicator strain (Fig. 7). Hence, maximum community diversity was obtained with low proportions of enterocin producers.

**Table 6:** General linear models on effects of the presence/absence of *Enterococcus*-bacteriocin producers on alpha diversity indices of the bacterial communities of cheeses

	d.f	*F*	*p*
Shannon diversity index	1;11	6.12	**0.031**
Faith’s phylogenetic diversity index	1;11	1.90	0.196
AVS richness	1;11	3.74	0.079

*d.f.* degree of freedom

The first number is the d.f. of the independent variable and the second is the d.f. of the error term. Significant *p*-value (*p* < 0.05) highlighted in bold

**Table 7:** Pearson’s correlation analysis results for the effects of proportion of *Enterococcus*-bacteriocin producers in two indicator strains on alpha diversity indices of the bacterial communities of the cheeses

Indicator strain		Shannon	Faith	ASV richness
*L. innocua*	*r*	− 0.46	− 0.56	− 0.49
	*p*	0.209	0.115	0.179
*E. faecalis* S47	*r*	− 0.65	− 0.58	− 0.40
	*p*	**0.041**	0.079	0.256

Significant *p*-values (*p* < 0.05) are shown in bold

**Table 8:** Correlation between the proportion of enterococci that are bacteriocin producers and alpha diversity indices of the *Enterococcus* populations per cheeses

Sensitive strain		Faith	ST-richness
*L. innocua*	*r*	− 0.64	0
	*p*	0.061	0
*E. faecalis* S47	*r*	− 0.28	− 0.56
	*p*	0.43	0.094

Dots represent the average, while boxes show the standard error of the mean and whiskers the confidence interval.

## Discussion

Cheese is a good model for the study of interacting multi-species microbial communities of reduced complexity [47], enabling comparisons with less manageable systems in which numerous bacterial species coexist. The microbial communities in cheese can be readily cultivated and managed, facilitating study of the patterns and mechanisms underlying the assembly, function, and interactions among constituents of microbial communities [48]. This study reveals a general trend towards greater diversity in LAB communities that include enterocin producers, although the greatest diversity is found when enterococcal abundance of enterocin producers are low to moderate. According to these findings, low levels of enterocin producers in enterococcal populations increase LAB diversity in cheese microbial communities.

LAB play an essential role in traditional cheese making by contributing to the development of its sensory characteristics and nutritional value [2]. LAB counts ranged from 2.8 × 10^5^ to 2.6 × 10^8^ in the cheeses under study (Fig. 1, Supplementary Table S1), within the range of values reported by other authors [49]. Massive DNA sequencing has shown that the main LAB genera in raw milk cheeses are *Lactococcus*, *Lactobacillus*, *Enterococcus*, *Streptococcus*, and *Leuconostoc* [50–53], as observed in the present cheese samples (Table 2 and Fig. 2). It is worth noting that, in one sample, (AM1902QU05) enterococci were detected by massive sequencing but not by a culture approach, while on the other hand, in two samples (AM1902QU08 and AM1902QU10), enterococci were detected by a culture approach, but not by metagenomic sequencing. While this discrepancy affected only a few samples in our study, it is frequent between culture-dependent and culture-independent approaches. The first case could be the result of dead cells or a viable but nonculturable (VBNC) state of these bacteria [54, 55], while the second case could be the result of primer bias or primer preferential annealing to other taxa [56, 57]*.*

*Enterococcus* is an important genus in LAB frequently found in traditional raw milk cheeses [58–61]. The proportion of enterococci can change during the cheese making process, being reported as between 10^4^ and 10^6^ CFU/g at the beginning of ripening and between 10^5^ and 10^7^ CFU/g at the end [62]. In the present samples, enterococcal counts ranged from 6.6 × 10^2^ to 1.1 × 10^6^ CFU/g (Fig. 1, Supplementary Table S1). The most frequently isolated enterococci were *E. faecalis* and *E. faecium*, followed by *E. durans* and *E. hirae* (Fig. 3), in agreement with previous reports on the greater abundance of *E. faecium* and *E. faecalis,* with a lesser amount of *E. durans* [63–67]. In addition to its proteolytic and lipolytic activities [22], enterococcal strains can also produce enterocins, which have the capacity to inhibit bacteria other than enterococci, including the pathogens *Listeria* and *Clostridium* [22, 68, 69]. Most of the present cheese samples contained enterococci that produced antimicrobial compounds (Fig. 4 and Table 3), and 21.2% of them demonstrated antimicrobial activity against both sensitive strains (*E. faecalis and L. innocua*) within the range described in previous studies [23, 70–72]. Moreover, 24.6% of the present samples evidenced antimicrobial activity against *L. innocua*. This type of bacteriocins is of particular interest in the cheese industry due to its ability to inhibit *Listeria* while allowing LAB to carry out their cheese ripening functions [69, 73].

The highly variable and complex nature of the microbiota in cheese makes competitive interactions inevitable, given that its members share the same niche and seek the same resources [47], which may ultimately affect the relative proportions of community members. Studies of cheese have shown that bacteriophage predation ensure bacterial diversity by eliminating large numbers of strains in accordance with the ‘kill the winner’ theory, thereby stabilizing the overall function of the community [74]. This theory, analogous to the classical Lotka-Volterra explanation of predator–prey population dynamics, proposes that a rise in the population of a host organism (the winner) in phage–bacteria interactions often results in a higher number of its corresponding predators (phages), increasing the kill rate of the winner [75]. Phages can have a drastic impact on fermentations that need LAB populations to reach high cell counts within a short time period [76]. In the present study, the presence of *Enterococcus*-enterocin producers significantly affected the Shannon diversity index of the community (Fig. 6 and Table 6), suggesting that species abundance in cheese is more equitably distributed when the community contains at least one producer. The impact of bacteriocinogenic strains depends on their ability to grow and produce optimal levels of bacteriocins, not only under technological conditions but also in the natural environment, where they must compete with other microbes in the food without altering its physicochemical or organoleptic characteristics [77]. In this way, the production of bacteriocins by NSLAB can have a positive effect by acting on other LAB strains such as starter cultures, promoting their lysis and release of enzymes that can contribute to the ripening process and enhance the flavor of the mature cheese [78].

Simulation studies have revealed that antagonistic interactions markedly increase the diversity and stability of a microbial community by promoting spatial structuring, which generates more robust populations [19, 79, 80]. In contrast, communities with a predominance of cooperative interactions are more easily destabilized [81]. This is because cooperation enhances between-species dependency, so that if one species decreases in abundance, it will tend to reduce the abundance of other species, destabilizing the system.

It has been proposed that antagonistic systems can promote diversity in environments under certain conditions, as experimentally demonstrated for *E. coli* colicin [19, 82]. However, conditions cannot always be controlled in natural environments. In a study of mouse gut microbiota, Umu et al. [83] found that dietary supplementation with Class II bacteriocin-producing bacteria had no effect on the overall structure of the community. Nevertheless, some significant alterations were observed at lower taxonomic levels, especially when the bacteriocins had relatively wide inhibitory spectra (e.g., enterocins Q and L50, and garvicin ML). The presence of bacteria-producing bacteriocins also increased the proportion of LAB, indicating the possibility of manipulating specific populations by treatments with bacteriocin producers at different levels and in different directions without affecting the normal inhabitants of the intestine. Qiao et al. [84] studied the effect of bacteriocin-producing *Pediococcus acidilactici* strains on the gut microbiota of normal mice, observing an increase in the total number of species but a slight decrease in the diversity and uniformity of the community. Likewise, only minor alterations in overall community composition were observed with ABP-118, a broad-spectrum class IIb bacteriocin produced by gut isolates of *Ligilactobacillus salivarius* [85]. In contrast, the present study focused on enterocin-producing populations in the community rather than a specific bacteriocin, considering bacteriocin production as an ecological factor in microbial interactions.

According to the present findings, bacteriocin production does not appear to affect the enterococcal population, with its effect being observed at community (between-species) but not population (within-species) level. This may be attributable to the highly variable spectrum of action of bacteriocins, which ranges from narrow to broad inhibitory activity against both closely-related and non-related species [86]. Broad-spectrum bacteriocins are more likely to be produced when the producer strain is highly abundant, allowing its ecological dominance to be consolidated through the suppression of community members in general [87]. For their part, narrow-spectrum bacteriocins are more focused on inhibiting the growth of competing strains that pose the greatest threat [87]. This is because closely-related species are more likely to exploit similar niches in an environment and engage in greater competition for space and resources [88]. In the present study, the bacteriocins may have a more specific spectrum of action against a genus other than *Enterococcus*. It is also possible that the producer strains are closely related (Fig. 3) and may share immunity mechanisms, increasing resistance to the antimicrobials being produced without affecting the diversity of the *Enterococcus* population [89, 90]. These types of ecological units of production and resistance have been described in other wild habitats [91]. Although we found no statistically significant variation in sensitivity between enterococci from the same cheese and those from different cheeses, a close-to-significant difference was observed (*p* = 0.068), suggesting the possibility of a greater degree of resistance from coexisting populations (Fig. 5).

In addition, the spatial structure is a key factor in promoting biodiversity by bacteriocin production. A spatial structure allows a producer to outcompete a sensitive strain even at much lower abundance levels because the bacteriocin does not diffuse away and reach a higher local concentration [19]. In this sense, cheese is a three-dimensional particulate gel matrix of casein or paracasein that wraps fats, minerals, and water [92]. Therefore, in this scenario, low abundant producers, such as the overserved here for enterocin producers, can have an impact on the overall community diversity due to the spatial structuring of producer populations.

In the present study, the presence of bacteriocin producers in the cheese microbiota was found to increase the bacterial diversity of cheese. However, examination of cheeses containing at least one producer revealed a negative correlation between diversity and the percentage of enterocin producers (Fig. 7). In other words, the existence of producers in the community increases its diversity, but this can be decreased by the presence of too many. In this way, the production of moderate bacteriocin levels may promote diversity by preventing one species from competitively excluding others [21]. These results are also compatible with the intermediate disturbance hypothesis, which proposes that species diversity is maximal at intermediate levels of disturbance, being sufficiently infrequent to allow many species to survive fluctuations but sufficiently frequent to avoid domination by more competitive species [93]; however, this hypothesis remains highly controversial [93–96].

Microbial community composition depends on biotic and abiotic factors. While physico-chemical factors are the first constraint to microbial development, microbial interactions fine-tune the microbial assemblage. Our findings suggest that bacteriocins not only play a role in the local structuring of resistant and sensitive producer strains but may also exert modulatory effects on whole microbial communities. It remains unknown whether these results can be extrapolated to other LAB species or even other wild microbial ecosystems. In this study, bacteriocin production proved to be important in controlling LAB communities during cheese ripening; further research is required on the effect of bacteriocin production on microbial diversity in other LAB communities and in wild microbial ecosystems.

## Supplementary Information

Below is the link to the electronic supplementary material.

Supplementary file1 (DOCX 16 KB)

Supplementary file2 (XLSX 27.4 KB)

## Data Availability

Sequence data are available at NCBI under the PRJNA988471 BioProject, including BioSamples from SAMN36020620 to SAMN36020683.
