# Supplementary material for: Bacteriocin-Producing Enterococci Modulate Cheese Microbial Diversity
Source: Microb Ecol. 2025 Jan 22;87(1):175. doi: 10.1007/s00248-025-02491-7 (PMC11750935; doi:10.1007/s00248-025-02491-7)
Supplement: Supplementary file 1 — Supplementary file1 (DOCX 16 KB) [file 248_2025_2491_MOESM1_ESM.docx]

**Supplementary Data**

**Supplementary Table S1. Main raw milk cheese features and microbiological counts.**

| **ID** | **Cheese** | **Milk type** | **Lactic Acid Bacteria**  **log(cfu/g)** | **Enterococci**  **log(cfu/g)** |
| --- | --- | --- | --- | --- |
| AM1901QU01 | Idiazabal | Sheep | 7.98 | 4.87 |
| AM1901QU02 | Pago de los Vivales | Sheep | 8.41 | 2.82 |
| AM1902QU03 | Pata de Mulo | Sheep | 6.16 | 4.21 |
| AM1902QU04 | Ardi Gazta Ekia | Sheep | 6.86 | 4.74 |
| AM1902QU05 | Comté, queso graso | Cow | 5.94 | ND^*^ |
| AM1902QU07 | Quesería Cortijo Júrtiga | Goat | 8.05 | 6.05 |
| AM1902QU08 | Quesería 3 RRR | Goat | 7.42 | 4.32 |
| AM1902QU09 | Granja Maravillas | Cow | 7.37 | 3.96 |
| AM1902QU10 | Granja Maravillas | Cow | 7.51 | 4.77 |
| AM1902QU11 | Emmental | Cow | 7.42 | 2.92 |
| AM1902QU12 | Le Moulis | Cow | 7.11 | 3.80 |
| AM1903QU14 | Las R.R.R. | Goat | 6.35 | 3.23 |
| AM1903QU15 | Cueva de la Magaha | Goat | 5.44 | 2.99 |
| AM1903QU16 | Mariscal | Sheep | 5.85 | 4.34 |
| AM1903QU17 | Las Abadías | Goat | 8.23 | ND^*^ |

^*^ Not Detected
